# Performance of homogeneous catalysts viewed in dynamics

**DOI:** 10.1039/d2cc05625a

**Published:** 2023-01-13

**Authors:** Wenjun Yang, Georgy A. Filonenko, Evgeny A. Pidko

**Affiliations:** a Inorganic Systems Engineering group, Department of Chemical Engineering, Faculty of Applied Sciences, Delft University of Technology, Van der Maasweg 9 2629 HZ Delft The Netherlands G.A.Filonenko@tudelft.nl E.A.Pidko@tudelft.nl

## Abstract

Effective assessment of catalytic performance is the foundation for the rational design and development of new catalysts with superior performance. The ubiquitous screening/optimization studies use reaction yields as the sole performance metric in an approach that often neglects the complexity of the catalytic system and intrinsic reactivities of the catalysts. Using an example of hydrogenation catalysis, we examine the transient behavior of catalysts that are often encountered in activation, deactivation and catalytic turnover processes. Each of these processes and the reaction environment in which they take place are gradually shown to determine the real-time catalyst speciation and the resulting kinetics of the overall catalytic reaction. As a result, the catalyst performance becomes a complex and time-dependent metric defined by multiple descriptors apart from the reaction yield. This behaviour is not limited to hydrogenation catalysis and affects various catalytic transformations. In this feature article, we discuss these catalytically relevant descriptors in an attempt to arrive at a comprehensive depiction of catalytic performance.

## Abstract 2

In this feature article, we discuss how the different aspects of the complex catalytic mechanisms manifest themselves in the commonly accepted performance metrics.

## Graphical abstract

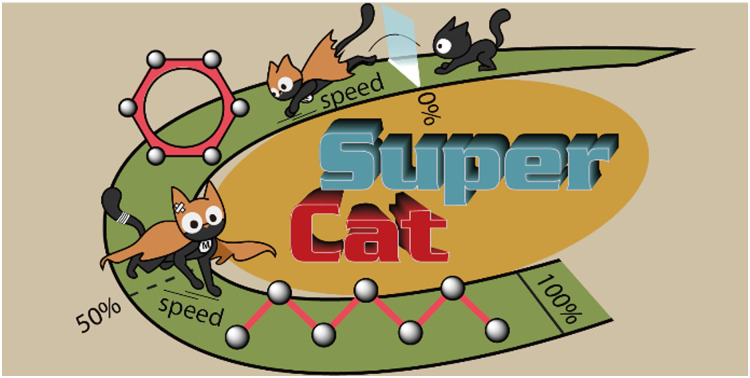

## 1. Introduction

### 1.1 The catalytic performance

Catalysis is at the heart of the modern chemical industry. The innovations in catalysis, *i.e.* the design and development of new superior catalysts, are constantly spurred by the new challenges faced in the manufacturing of chemicals. These are the increasing demand for fine chemicals, the shortage of fossil fuels, and the new environmental regulations limiting the use of toxic reagents. As a result, the modern assessment of catalysts is multidimensional. Apart from the traditional terms of activity, selectivity and stability, new metrics for catalysts including sustainability, environmental impact, toxicity as well as biocompatibility are gradually taking root.^1–4^ Therefore, when evaluating catalysts one deals with a complex product that is not strictly defined. In this perspective, the main focus of catalyst assessment will be on, presumably, much less complex and more generally accepted metrics such as catalytic performance.

**Fig. 1:**
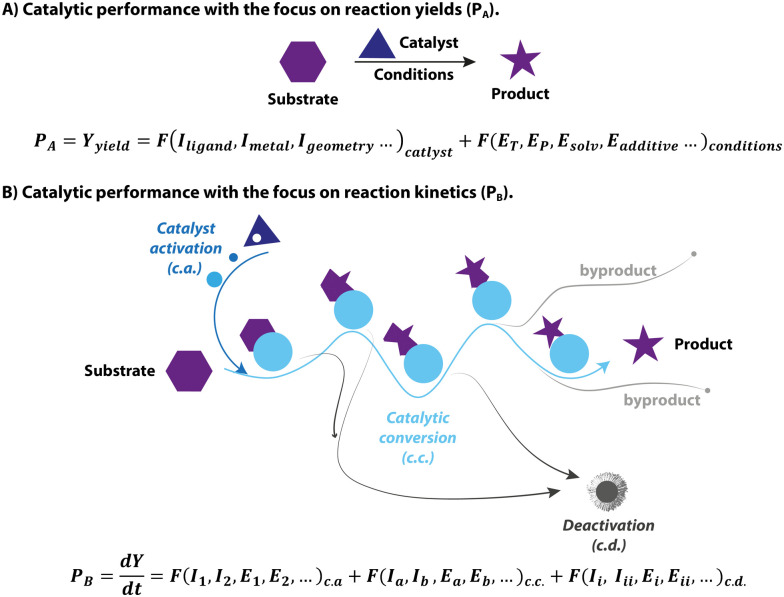
Two different views of the catalytic system and corresponding describing methodologies of catalytic performance (*P*) with catalyst structural parameters (*I*) and reaction condition parameters (*E*).

The yield of a catalytic reaction is often used as a primary metric of catalytic performance in organic chemistry and homogeneous catalysis communities. The typical approach for finding an active homogeneous catalyst usually relies on trial and error in an attempt to reach a high yield. Specifically, the modern high-throughput catalyst screening begins with the formulation of massive libraries of transition-metal/ligand combinations. These catalyst combinations are then rapidly screened for a model chemical conversion under pre-defined conditions to pick out the best performing, in terms of the yield of the target product, candidates, for which the operating conditions will be further optimized. In this framework, catalysis is treated as an elementary reaction, and the final yield is equal to the performance of the catalyst (Fig. 1A). However, catalysis as a phenomenon is centred around the acceleration of chemical transformation, *i.e.* improvements in kinetic behaviour, not the yield itself. From the catalyst perspective, the final yield of a catalytic transformation depends on a balance between the rates of the target reaction and various side-processes resulting in the selectivity or active species loss (Fig. 1B). While most screening studies aimed at enhancing the final yield by varying the catalyst structure and reaction environment, they often fall short of providing kinetic data that describes catalysis in universal terms.

In addition to the mismatch between the yield terms used in conventional optimization and the kinetic nature of catalysis, the complexity of catalytic systems can also pose a significant challenge to studying the intrinsic activity of the catalysts. The latter becomes evident owing to the rapid development of advanced experimental techniques, which allow unprecedented insight into the molecular behaviour of a catalyst as it undergoes distinct stages of catalyst activation, deactivation and reaction turnover (Fig. 1B). These processes comprise the complex catalytic reactivity network that defines the time-dependent catalyst speciation under specified conditions. Growing evidence in the literature reveals that apart from the catalytic turnover, other processes can also impact the apparent kinetics and outcome of the system. As a result, there can be profound distinctions between catalytic performance and intrinsic activity of catalysts, making it elusive and challenging to study. This phenomenon is a continuing research interest of our group, and in this feature article, we showcase the impact of complex catalysis networks and the ambiguity of common metrics of catalytic performance on homogeneous hydrogenation.

### 1.2 Molecular catalysts for reductive transformations

Reduction reactions, and those of carbonyl compounds specifically, are essential chemical transformations with far-reaching applications in industry for producing pharmaceuticals, fragrances and other fine-chemical intermediates.^5^ This field has progressed from stoichiometric reductions to modern hydrogenation catalysis, which circumvents the generation of considerable amounts of waste.^6^ Heterogeneous catalysts are the work-horse of industrial hydrogenation.^7,8^ Despite the harsh conditions commonly required for the operation, they are widely employed for the production of bulk chemicals where selectivity is not a primary concern. Molecularly-defined homogeneous transition metal-based catalysts take a role of a complementary tool for lower-temperature and more “specialized” catalytic reductions in synthetic applications requiring high selectivities and/or high tolerance of functional groups, commonly encountered in fine- and pharmaceutical chemistry industries.^9^

**Fig. 2:**
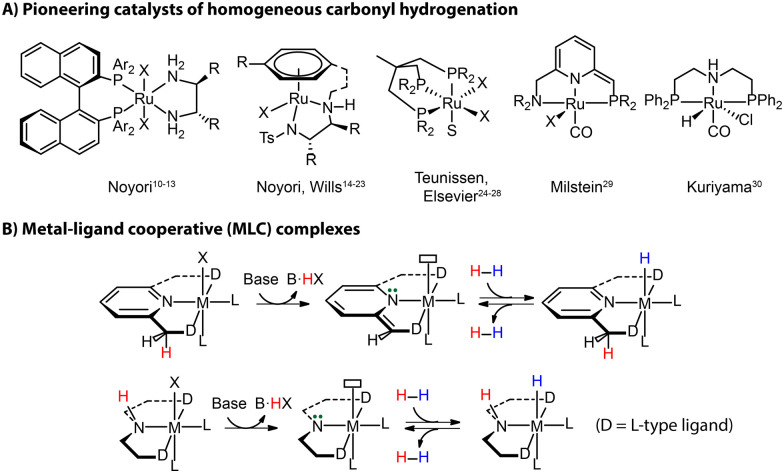
Landmarks of homogeneous hydrogenation (A) and the activation mode of metal–ligand cooperative (MLC) complexes *via* protonation and deprotonation (B).

The 2001 Nobel Prize in Chemistry has been awarded to Noyori for his work on asymmetric hydrogenation catalysts [(P^P)Ru(N^N)], which highlights the prominent role that hydrogenation techniques acquired in the modern industry (Fig. 2A).^10–13^ Other seminal landmarks include the powerful [(N^N)Ru(arene)] catalyst by Noyori and Wills for the efficient asymmetric carbonyl transfer hydrogenation,^14–23^ the robust Ru(Triphos) catalyst by Teunissen and Elsevier that turn out to be effective for the hydrogenation of challenging carboxylic acid substrates,^24–28^ the lutidine-based Ru(PNN) complex by Milstein,^29^ and the Ru-MACHO family of complexes by Kuriyama^30^ as the inspiration of the bifunctional ester hydrogenation catalysts (Fig. 2A).

The vast majority of efficient carbonyl hydrogenation catalysts have been bifunctional complexes featuring an acidic moiety in the ligand backbones (Fig. 2B).^31^ This reactive site can be deprotonated with an external base to generate the reactive molecular system composed of a highly basic site on the ligand and a Lewis acidic metal center. This acid–base pair is able to split H_2_ heterolytically to produce a metal hydride and adjacent Brønsted acid sites, which can reduce carbonyl compounds *via* a concerted/cooperative hydride transfer and protonation. Such a process represents a typical metal–ligand cooperation (MLC) mechanism based on protonation/deprotonation (Fig. 2B). While the degree to which the MLC is involved in catalysis remains debated,^32,33^ the introduction of ligand platforms with cooperative sites has been confirmed as a versatile way to induce hydrogenation reactivity in transition metal catalysts.^34,35^

Significant progress has been made in this field mainly by noble metal complexes based on ruthenium, iridium, rhodium, and osmium.^36–39^ The requirements for more sustainable processes recently also initiated an intensive investigation of alternative catalysts based on earth-abundant, inexpensive 3d metals (Fe, Co, and Mn).^35,40–46^ Taking this idea a step further, the transition metal-free catalytic hydrogenation was achieved in the past decade by ‘frustrated Lewis pair’ catalysts but is still in its infancy.^47–54^ Although a great amount of hydrogenation catalysts has been developed, understanding the fundamental laws of hydrogenation catalysis that would allow for designing new catalytic systems rationally, remains a central subject in modern catalysis research.

In the following sections, we discuss the effects that pre-catalyst activation, catalyst deactivation and the reaction environment have on hydrogenation catalysis. By linking the catalytic performance to the transient behavior of the catalyst in these processes, we can reveal its highly dynamic, time- and condition-dependent nature that is far too complex to rely on final yield as a single descriptor of the performance. New descriptors for each catalytic stage will be discussed together with their role in establishing a more comprehensive description of catalytic systems. We argue that the high complexity of the common catalytic systems and the pronounced condition-dependence of their behavior may result in the failure of the common catalyst evaluation strategies based on the integral yield in their primary objective, that is to probe the relation between the molecular catalyst (metal–ligand combination or isolated organometallic pre-catalyst) and its catalytic performance.

## 2. Pre-catalyst and its activation

The extreme sensitivity of active catalytic species renders their isolation and direct use in catalysis arguably complicated, if not impossible. Because of this, chemists often make use of pre-catalysts, which transform to the active state either *via* a separate activation procedure or *in situ* within the reactive mixture. In the context of this article, we will use the term “catalyst activation” to describe the process of conversion of the pre-catalyst to the catalytically active state, *i.e.* the entry into the catalytic cycle. By extension, catalyst deactivation would embody a set of transformations, by which the catalyst can leave the catalytic cycle. While the pre-catalyst activation takes place outside of the catalytic cycle, it can still be an important factor that affects overall catalyst performance. As with every chemical transformation, catalyst activation can proceed with varying rates and selectivity, both capable of affecting the outcome of the catalytic process as a whole.

### 2.1 The rate of pre-catalyst activation

The rate of pre-catalyst activation defines how quickly the active species or their precursors are supplied to the catalytic cycle. Depending on the activator and conditions, this supply can either be instant or slow in time, resulting in the different effective concentrations of the active species in the reaction mixture and, therefore, different reaction rates.

**Fig. 3:**
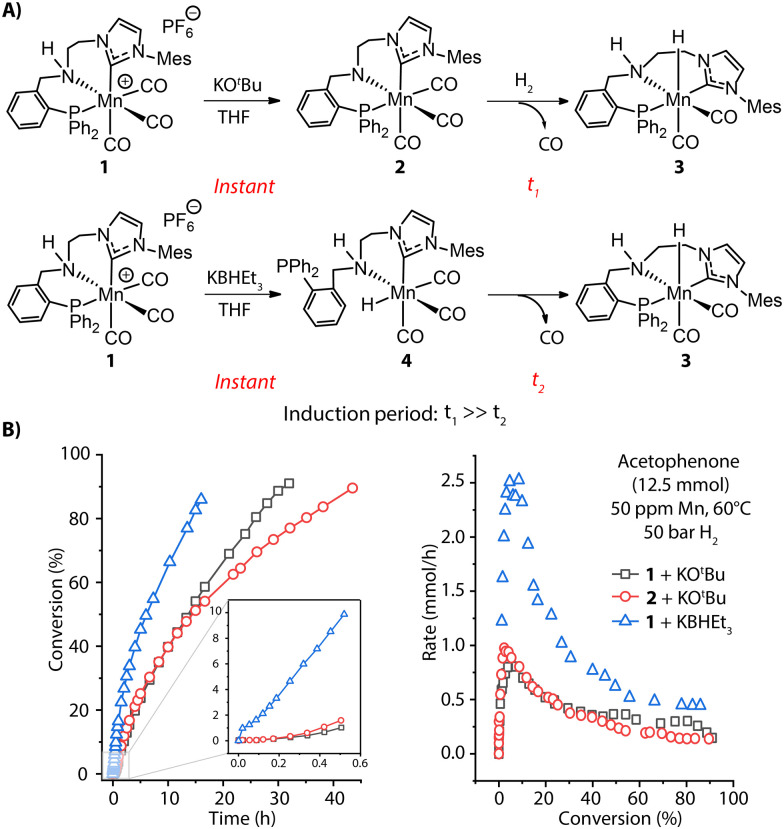
Activation of MnCNP pre-catalysts and corresponding catalytic performance. (A) Generation of active Mn hydride 3 through CO dissociation with two different activation protocols: alkoxide base KO^*t*^Bu and hydride donor KBHEt_3_; (B) the conversion and rate profiles for the hydrogenation of acetophenone with 1 and 2 in the presence of 1 mol% KO^*t*^Bu or 1 mol% KHBEt_3_ promoters at 60 °C, 50 bar H_2_ and 50 ppm Mn loading. Adapted with permission from ref. 55.

Apart from base-induced dehydrohalogenation (Fig. 2B), known as a rapid, nearly instantaneous process, bifunctional hydrogenation pre-catalysts may require further transformations during activation, *e.g.* dissociation of an ancillary ligand to open the coordination site for hosting substrate molecules. Such processes are quite common in transition-metal catalysis and can become the rate-determining step for the activation. For example, the highly active ketone hydrogenation Mn-CNP (1) pre-catalyst recently developed by our group is a cationic complex and readily offers a coordinatively-saturated amido complex 2 under basic conditions.^55^ One of the three carbonyl groups in 2 needs to be detached before H_2_ can be split to generate the Mn-hydride species and enter the catalytic turnover. This transformation is very sluggish, evidenced by the slow replacement of CO with hydride in a stoichiometric experiment of 2 as well as the long induction period observed for the catalytic reaction with both 1 and 2 (Fig. 3A, top and B, left). Alternatively, the treatment of pre-catalyst 1 with KBHEt_3_ readily furnishes 3*via* the Mn hydride 4 featuring a free phosphine arm (Fig. 3A, bottom). The reattachment of strong P donor facilitates the CO dissociation and accelerates greatly the formation of the catalytic complex 3, resulting in a higher overall catalytic performance. This is evident from the rate profile presented in Fig. 3B showing that the improved activation protocol allows achieving a 2.5-fold higher reaction rate with formally the same catalyst concentration. The sluggish catalyst activation can hamper the overall catalytic performance, obscuring part of the intrinsic activity of the examined catalyst.

**Fig. 4:**
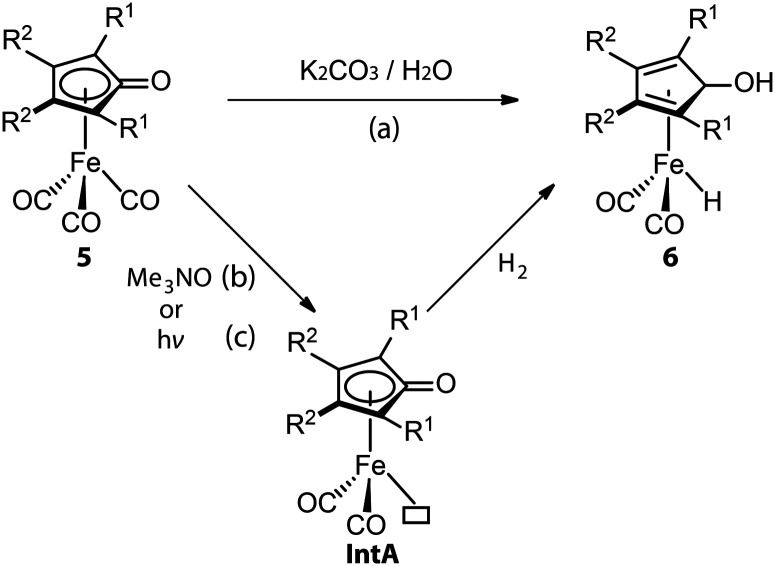
Removal of CO ligand in the activations of (cyclopentadienone)iron carbonyl complexes. (a) Conversion of Fe-CO to Fe-hydride *via* a Hieber reaction; (b and c) generation of vacant coordination sites *via* UV-induced CO dissociation or oxidative cleavage of CO with Me_3_NO followed by the activation of H_2_.

Similarly, CO dissociation is the key step for the activation of Knölker-type iron complexes (5, Fig. 4). While these complexes were first reported in 1953,^56–59^ their catalytic applications for hydrogenation remained unknown until the effective activation, conversion of a CO ligand to hydride, was achieved *via* a Hieber reaction (Fig. 4, path a).^60,61^ In principle, the iron hydride is also accessible *via* the direct thermal dissociation of CO ligand under H_2_, which however is kinetically challenging. This pathway was later enabled by powerful oxidative cleavage with Me_3_NO or UV irradiation (Fig. 4, paths b and c).^62^ The resulting intermediate IntA can activate H_2_*via* an MLC mechanism to yield the Fe hydride species 6. Due to the high sensitivity of 6, 5 is generally utilized as the pre-catalyst for hydrogenation and requires an *in situ* activation.^63–68^ The rate of catalyst activation *via* a particular approach can determine the performance of 5. However, systematic kinetic studies on different activation protocols or the catalysis initiated *via* them are rarely carried out,^69–71^ as is also the case for these systems. Protocols (b) and (c) are presumably much faster, resulting in generally better catalytic results that utilize them.^72,73^ The activation protocol with Me_3_NO, however, is more widely used in hydrogenations because of its higher compatibility with pressurized reactors. Based on the understanding of the activation process, a more-labile nitrile ligand was introduced to replace one of the CO ligands in 5, furnishing the new pre-catalyst that can achieve the activation-free transfer hydrogenation of aldehydes and ketones.^74^

**Fig. 5:**
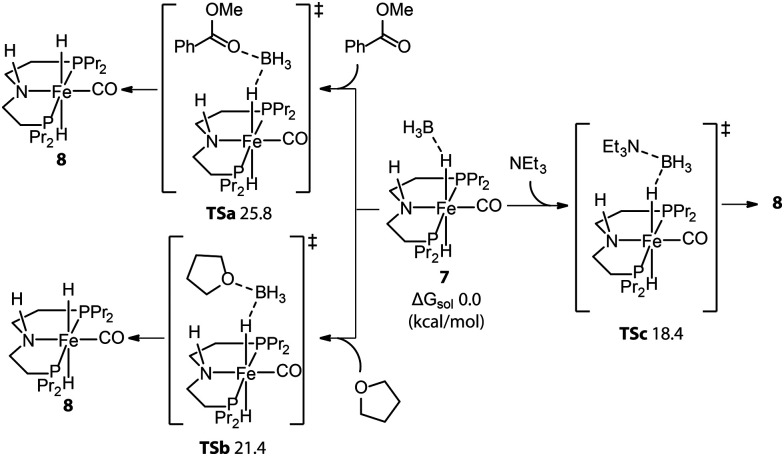
Trapping BH_3_ from the Fe-PNP pre-catalyst. The energetics for the generation of active catalyst species 8*via* the removal of BH_3_ with different Lewis bases: ester substrates, tetrahydrofuran solvents, and trimethylamine additives. Adapted with permission from ref. 78. Copyright (2014) American Chemical Society.

Even molecularly defined metal hydrides may require an additional activation for catalysis. For example, the Fe-PNP hydride species 7 was found to be a BH_3_ adduct by Hazari and Schneider.^75^ Beller^76^ and Guan^77^ independently developed the efficient base-free ester hydrogenation with this well-defined pincer complex. Mechanistic studies demonstrated that the dihydride complex 8 is the catalytically active species for these reactions, while the dissociation of BH_3_ is essential for the initiation of pre-catalyst 7. The use of a Lewis base is a straightforward way to trap the Lewis acidic BH_3_. To study how 8 forms under the catalytic conditions, Guan employed density functional theory (DFT) to compute the energetics for the BH_3_ trapping process with different Lewis bases, such as an ester substrate, a tetrahydrofuran solvent, or a trimethylamine additive (Fig. 5).^78^ DFT calculations showed that the activation of 7 with the strong Lewis base Et_3_N was the fastest process with the lowest energy barrier (18.4 kcal mol^−1^). Indeed the rapid catalyst activation with the Et_3_N promotor was reflected in a nearly twice higher rate of the ester hydrogenation compared to the control experiment without additives.

**Fig. 6:**
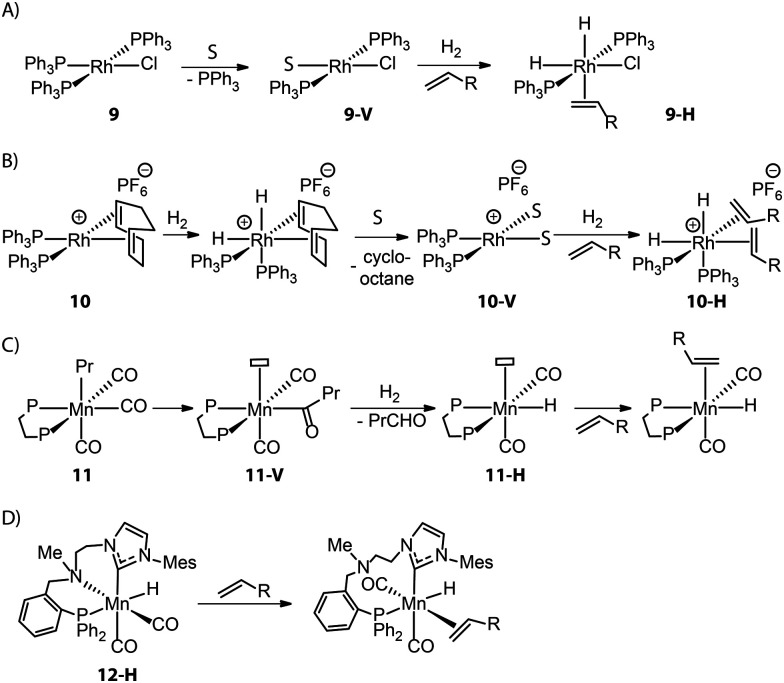
Different strategies for generating vacant sites in the activations of alkene hydrogenation/transposition catalysts. Note: S represents solvent molecule. (A) Direct dissociations of phosphine ligands in Wilkinson's catalyst 9; (B) reduction of diene ligands followed by the rapid dissociation in Schrock–Osborn catalyst 10; (C) consumption of CO ligand *via* its migratory insertion to Mn–alkyl followed by hydrogenolysis in Mn alkyl catalysts 11; (D) the temporary dissociation of dynamic ligand in N-alkylated Mn-CNP catalyst 12-H.

Control over the catalyst activation chemistry is critical for catalysis in general. A classical example is Wilkinson's alkene hydrogenation catalyst (PPh_3_)_3_RhCl (9), which performance is limited by the slow dissociation of phosphine ligands (Fig. 6A).^79^ Ancillary diene ligands (*e.g.* 1,5-cyclooctadiene) that can readily dissociate after being hydrogenated and weakly coordinating anions that would remain in the second coordination sphere were introduced to accelerate the formation of adequate open sites (Fig. 6B). The resulting Schrock–Osborn catalyst (PPh_3_)_2_Rh(diene)[PF_6_^−^] (10) gave *ca.* 6-fold higher turnover frequency in the hydrogenation of terminal alkenes compared to the classical catalyst 9.^80,81^

Such a hydride transfer to olefins is more challenging for Mn(i) catalysts that are coordinatively saturated with high-field ligands. An ancillary CO ligand must be removed from the neutral Mn(i) complex to furnish a vacant site for the coordination of an olefin molecule next to the active Mn-hydride, which can be kinetically unfavorable. Kirchner and co-workers found that an Mn–alkyl complex readily furnished a 16e^−^ Mn hydride species 11-H*via* the migratory insertion of a CO ligand into an Mn–alkyl bond followed by hydrogenolysis (Fig. 6C).^82^ This coordinatively unsaturated Mn–H enabled the first Mn-catalyzed hydrogenation of alkenes. Alternatively, ligand dynamics can be utilized to temporarily create free space and initiate the catalytic turnover. As disclosed by our group, the alkylation of the N–H group within the Mn–CNP complex (1) could elevate the steric hindrance of the N donor and facilitate its reversible dissociation.^83^ This strategy led to the new catalyst 12-H which is highly efficient in transposition as well as hydrogenation of olefins (Fig. 6D).

The examples described above point out that the performance of catalysts in operation can be highly dependent on the rates of their activation protocols. For some hydrogenation systems, the dissociation of an ancillary ligand from the pre-catalyst, commonly treated as a simple process, can not only limit the rate of the catalyst activation but also determine the apparent performance of the catalytic system. An induction period observed in reaction kinetics is a strong indicator of slow activation. Detailed mechanistic and kinetic analysis of catalyst activation provides practical guidance on how to accelerate the pre-catalyst activation and significantly improve the catalytic results.

### 2.2 The selectivity of pre-catalyst activation

**Fig. 7:**
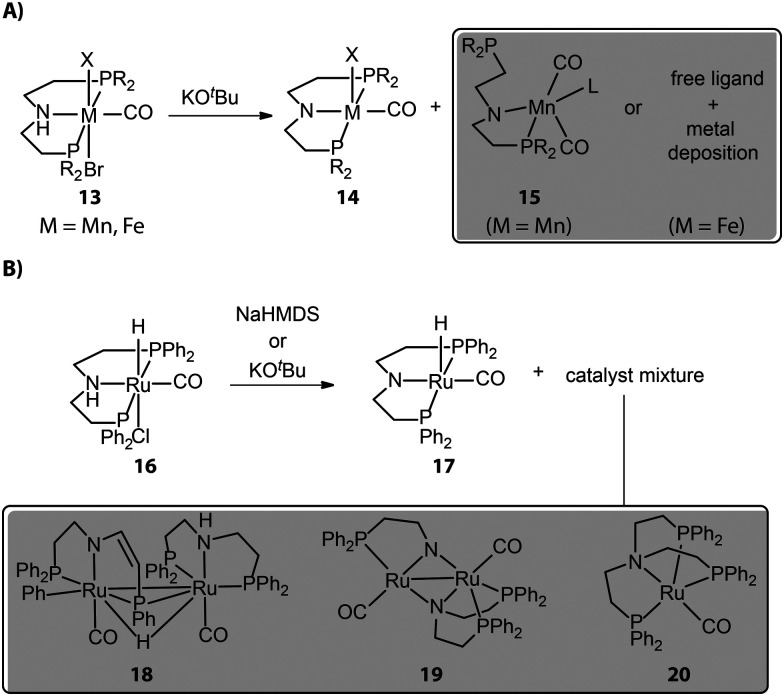
The degradation of hydrogenation pre-catalysts during the activation stage. (A) The reactions of catalysts based on first-row transition metals (Mn and Fe) with a strong base can lead to the partial or full dissociations of ligands. Note: the L of complex 15 can be Mn metal or any molecule of the activation system, *e.g.* solvent, *tert*-butoxide. (B) The base-activated Ru complex tends to form Ru dimers.

Most chemical reactions do not proceed with quantitative yields and selectivity, and those that cause catalyst activation are no exception. Reactions with organic or inorganic bases, generally required for activating bifunctional hydrogenation pre-catalysts, despite their formal simplicity can often lead to catalytically inert or less reactive side-products, causing partial catalyst degradation. For example, Mn complexes with typical aliphatic PNP pincer ligands show a multitude of reactions upon base activation. The reactions of such Mn bifunctional catalysts with strong bases are known to readily result in the active dicarbonyl amido complex 14 (Fig. 7A). However, the repulsive electrostatic force within the Mn complex is stronger compared to noble-metal ones due to the smaller ionic radius of the Mn center. As a result, Mn complexes should have low constraints of the coordination geometry and can collapse to the structures with lower coordination numbers.^84^ The pronounced covalence of the Mn–N bond in Mn species 14 could further enhance the ring strain of the complex as evidenced by its bipyramidal rather than square pyramidal geometry, leading to the dissociation of the side arm of the ligand.

Our group studied the direct activations of various Mn-MACHO (Et_2_, ^*i*^Pr_2_) with alkoxide bases and observed that in addition to the desired 14, small amounts of deprotonated Mn–PN complexes 15 featuring a dissociated P-donor were also generated.^85^ The deprotonated Fe–PNP complexes, analogous to Mn–PNP, were prone to fully collapse, giving only free pincer ligand and metal deposits as reported by Hazari and Schneider.^86^ As for the late-transition-metal hydrogenation catalysts, the coordination geometry of the base-activated species is typically stable; however, the presence of the vacant site in the square pyramidal activated complex may favor the complex aggregation and the formation of dimers. When tracking the activation of widely used Ru-MACHO (16), Schaub and co-workers observed the generation of Ru dimer complexes 18, 19, and a tripodal Ru(0) 20 apart from the target amido complex 17 (Fig. 7B).^87^ Apparently, the dimerization was the main degradation pathway of the catalyst. The bidentate PN in 19 and tetradentate NP_3_ in 20 also indicated the occurrence of disproportionation of the PNP ligand.

For Mn(i) catalysts, the 5-coordinated nature of the activated states creates a possibility for the effective equilibrium between the square pyramidal and trigonal pyramidal isomers. The latter may stabilize the excited triplet state, which opens a path towards one-electron oxidative or disproportionation degradation of the active Mn(i) complexes. Furthermore, the presence of activated carbonyl ligands within the defined molecular precursors or the common Mn(i) sources (Mn(CO)_5_Br, Mn(CO_3_)Cp*, etc) used for *in situ* catalysis may additionally complicate the activation procedure utilizing alkoxide base reagents. The alkoxide base may attack the Mn-bound CO ligand to form a reactive acyl intermediate.^88^ The expert-bias-free computational exploration of potential deactivation channels using graph-based reaction network analysis indicates that such reactivity may open a path towards partial ligand dissociation and the formation of low-coordinate species that may represent the onset of long-term catalyst deactivation.^89^

The loss of the catalyst during the activation stage typically leads to a permanent decrease in the catalytic performance. A thorough characterization of the side products formed during the activation is typically required to spot it. A common way to promote the selective supply of active catalysts is by introducing a donor that can stabilize the coordinatively unsaturated intermediate prior to the activation process. The substrates with carbonyl groups in hydrogenation catalysis are the candidates for such donors.

**Fig. 8:**
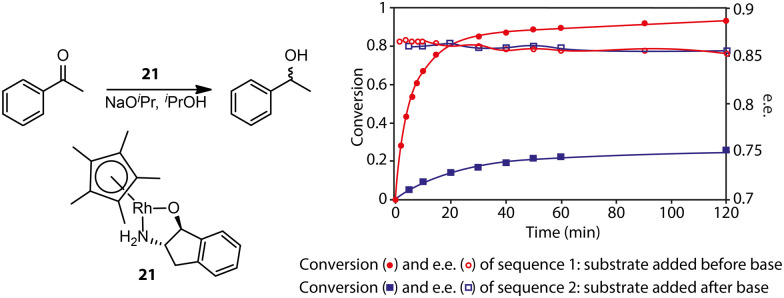
The effect of pre-catalyst degradation on the reaction kinetics and the stabilizing effect of carbonyl substrates on pre-catalyst activation. (Left) The transfer hydrogenation of acetophenones catalyzed by an Rh bifunctional catalyst 21. (Right) The conversion and enantiomeric excess (e.e.) kinetics for the transfer hydrogenation operated in two different procedures: sequence 1, ketone substrates were added before the activation of the pre-catalyst initialized by NaO^i^Pr; sequence 2, ketone substrates were added after the activation of pre-catalyst 21. Adapted with permission from ref. 90. Copyright (2004) American Chemical Society.

Gavriilidis and co-workers demonstrated that the addition of substrate before base activation can boost the performance for the asymmetric transfer hydrogenation of ketone catalyzed by bifunctional Rh/aminoindanol 21 (Fig. 8).^90^ The observed much lower rate from the onset of the reaction as well as the identical enantioselectivity in sequence 2 compared to sequence 1 confirmed the presence of a profound pre-catalyst degradation during the activation step. Apparently, the weak donor ligand, substrate acetophenone, can suppress such an unfavorable effect. For highly labile activated species, strong donors may be required for their stabilization. Monodentate phosphine ligands were reported as efficient additives for improving the selectivity of hydrogenation pre-catalyst activation.^91–94^ Schaub and co-workers observed improved performance in Ru-MACHO-catalyzed dehydrogenative coupling of hexanol with the addition of phosphine.^94^ It was possible that the improved performance of Ru-MACHO stemmed from the electron-donating ability of phosphine additive. A quantum-chemical study showed that the catalytic pathway with phosphine dissociation is energetically much more favorable, suggesting the main role of the phosphine additive is to stabilize the active catalyst.

As discussed above, the activation of hydrogenation pre-catalysts with a strong base can lead to their partial degradation. As a consequence, the observed catalytic performance does not correspond to the intrinsic activity of the catalyst, but rather to that of the remaining amount of active species after the activation. Care needs to be taken regarding the activation sequence, activation time as well as loading of the reactive compounds. The addition of stabilizers can be beneficial for highly sensitive catalysts. However not every undesired change during the activation process leads to degradation: on occasion, some ligand rearrangements and metal dimerization events are advantageous for catalysis, as discovered by groups of Chianese^92^ and Gusev.^95^

## 3. Reaction environment and its composition dynamics

Multiple components comprise catalytic systems. Apart from being reactants, stoichiometric reagents or catalyst species that are directly relevant for catalysis, the components of the reaction mixture contribute to defining the reaction environment indirectly. In principle, the efficiency of any given catalytic process is dependent on the reaction environment. Since catalysis involves the consumption of starting materials and the formation of new molecules, the activity of the catalyst in the course of the reaction would not be constant due to the change in reaction mixture composition. These effects should be more pronounced as reactant concentrations increase with the limit of the solvent-free processes. In the case of carbonyl hydrogenation, the reaction environment changes from aprotic to highly protic and polar one during the transformation. Such changes in conditions can strongly affect the behavior of the catalytic system.

**Fig. 9:**
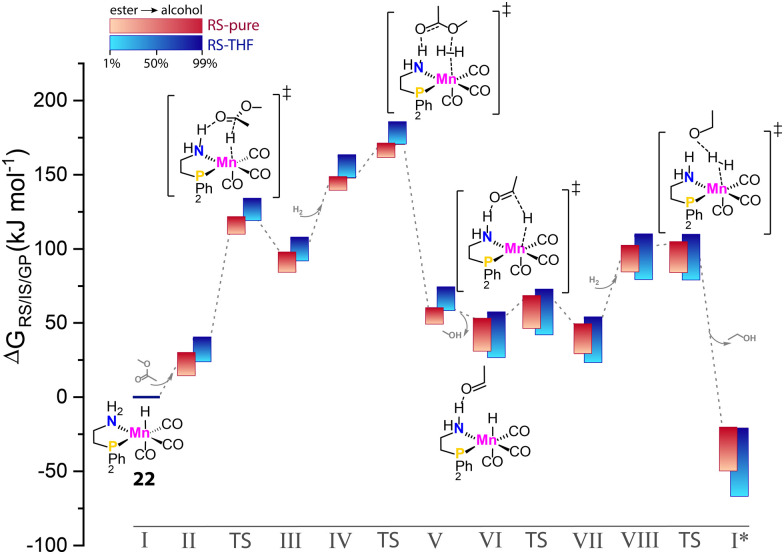
The dynamic energetics of catalysis in the course of reaction. RS-computed The operando free energy diagrams of Mn–PN-catalyzed ester hydrogenation along the reaction coordinate. Here only the hydrogen-shuttle catalytic pathways in THF (RS-THF) or neat condition (RS-pure) are shown. Adapted with permission from ref. 96. © Licensed under a CC BY-NC-ND 4.0 license.

Krieger and co-workers computed the operando free energy diagrams for the homogeneous ester hydrogenation with Mn–PN catalysts (22) in THF and neat conditions.^96^ Specifically, the energetics of individual states in the catalytic cycle accounted for the varied concentrations of the ester substrate and alcohol product *via* the COSMO-RS solvent model to mimic the evolution of the reaction mixture composition in the course of the reaction. It was found that the free energy surface underlying the catalytic cycle *via* a hydrogen shuttle mechanism was highly dynamic and conversion-dependent (Fig. 9). Note that this computational study did not consider side-reactions and catalyst deactivation paths, but focused solely on a single favorable catalytic mechanism. When the conversion-dependent changes of the reaction medium composition were taken into account, the energetics of each state increased to a different degree as the reaction progressed.

Catalysis under neat conditions exhibited more pronounced perturbation in the course of the reaction compared to the reaction in THF. In general, these changes caused a decrease in overall reaction favorability, *i.e.* thermodynamics, as the reaction proceeds. For some elementary steps, highly nonlinear behavior of their kinetic and thermodynamic parameters with the progress of the reaction was observed. This gave rise to the non-monotonous trends in the kinetic profile predicted by microkinetic modeling.

**Fig. 10:**
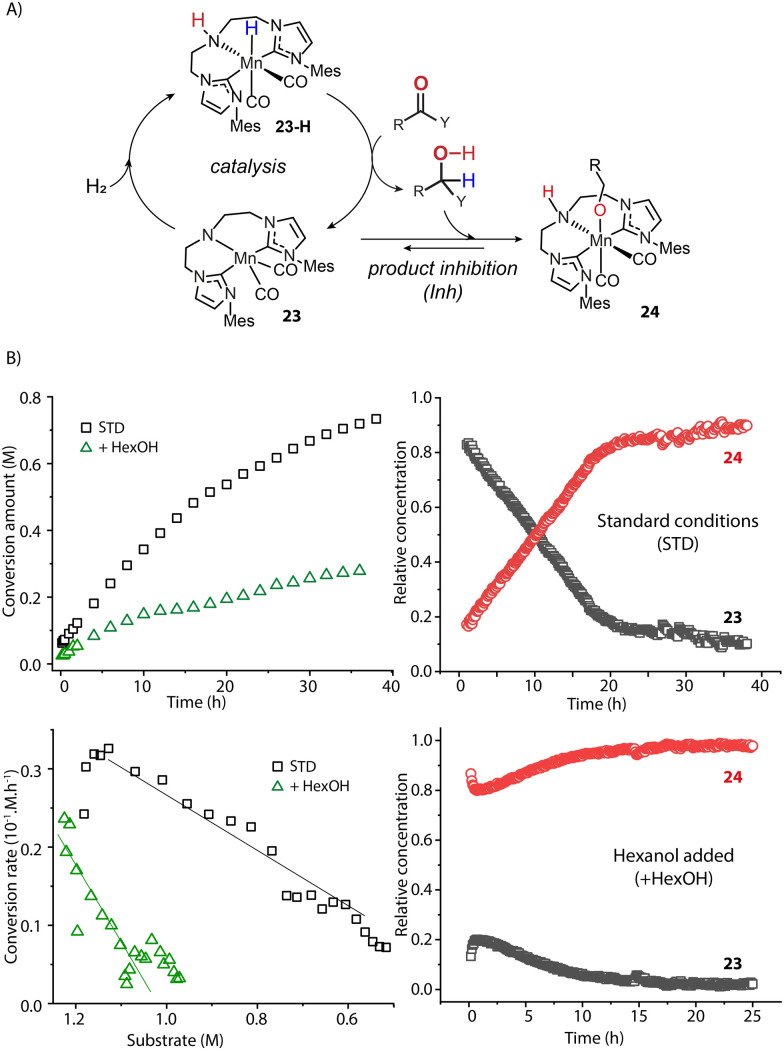
The impact of dynamic reaction environment on the performance of an ester hydrogenation catalytic system. (A) The catalytic cycle of the Mn-CNC-catalyzed ester reduction. The alcohol product can bind to 23 and form inhibited species 24; (B) the kinetics and reaction rate plots (left) as well as the real-time concentration of catalyst species (right) for the hydrogenations of hexyl hexanoates catalyzed by Mn-CNC. The catalyst evolution was traced by operando IR spectroscopy. Conditions - standard: hexyl hexanoate (1.25 M), catalyst (0.1 mol%), KO^*t*^Bu (10 mol%) in THF (8.2 mL), 70 °C, 40 bar H_2_; hexanol added: extra alcohol added at 1.25 M. Adapted with permission from ref. 97. © Licensed under a CC BY 4.0 license.

The catalyst speciation can also be dynamic in the course of the reaction. Our group showed this by the example of a Mn-CNC-catalyzed ester hydrogenation system where the alcohol product could further bind to the activated amido species 23 and form Mn-alkoxide 24 (Fig. 10A).^97^ Monitoring the hydrogenation with operando-IR spectroscopy revealed that state 23 comprised over 90% of the ‘catalytic’ Mn(i) species at the beginning and was likely the resting state in catalysis. However, along with the production of alcohol, the proportion of 23 continuously decreased with 24 becoming the dominant species eventually (Fig. 10B). A control experiment with the addition of extra alcohol prior to catalysis displayed a large proportion of Mn alkoxide and much lower catalytic rate from the onset of reaction, confirming the inhibitory effect of the product. Therefore the accumulation of alcohol products continuously consumed 23, leading to a severe drop in the steady state concentration of active catalyst species. As a result, the catalytic efficiency of ester hydrogenation was substantially inhibited. Given the numerous reports on the formation of stable metal alkoxides,^98–105^ we envision the product inhibition effects should be common in carbonyl hydrogenation systems. They should decrease the catalytic efficiency to a different degree depending on the thermodynamic stability of the inhibited state.^106^

The most straightforward way to suppress the inhibitory effect of the reaction product is to remove it during the catalysis, apart from varying the ligand structure and the nature of the catalyst.^88^ As reported by Hansen and Rosner, the *in situ* derivatization of amine product with the addition of di-*tert*-butyl dicarbonate ((Boc)_2_O) resulted in *ca.* 16-fold higher hydrogenation rate compared to the control experiment.^107^

**Fig. 11:**
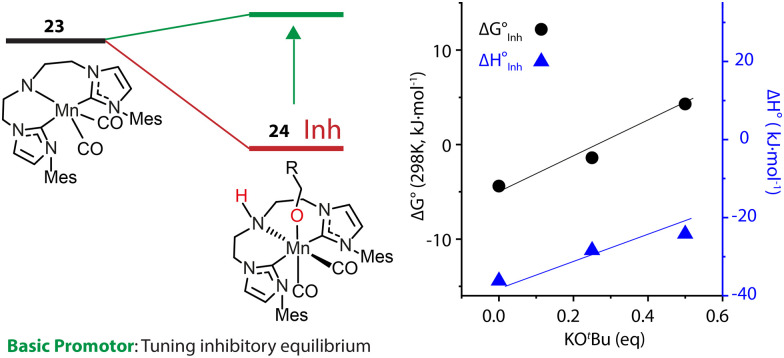
The dependence of the free energy surface for inhibitory equilibrium on base concentration. The Gibbs free energy changes and enthalpy changes of the equilibrium 23–24 at different concentrations of KO^*t*^Bu in THF. Adapted with permission from ref. 97. © Licensed under a CC BY 4.0 license.

An alternative approach is to tune the free energy surface of the inhibitory process by using its intrinsic condition-dependency. The studies from our group on Mn-catalyzed ester reduction pointed to the ability of alkoxide base additives to favor the product elimination from the Mn–OR adducts.^108,109^ For the Mn-CNC system (Fig. 10), we demonstrated that the increase of KO^*t*^Bu concentration significantly elevated the standard Gibbs free energy of alkoxide formation (23–24) from negative to positive and rendered this transformation unfavorable (Fig. 11). Accordingly the lifetime of the active catalyst in the high-base-loading experiment was prolonged, leading to the substantial enhancement of the efficiency of ester reduction.

The dependence of the standard free energy of such equilibrium on the base concentration was also observed for other Mn hydrogenation catalysts.^97^ Since the base promotor is formally not involved in the transformation, it can be viewed as a component of the reaction medium that perturbs the reaction environment for the inhibitory process. This was the first precedent of such indirect action on the intrinsic thermodynamics of elementary steps of catalysis resulting into pronounced condition-dependence.

**Fig. 12:**
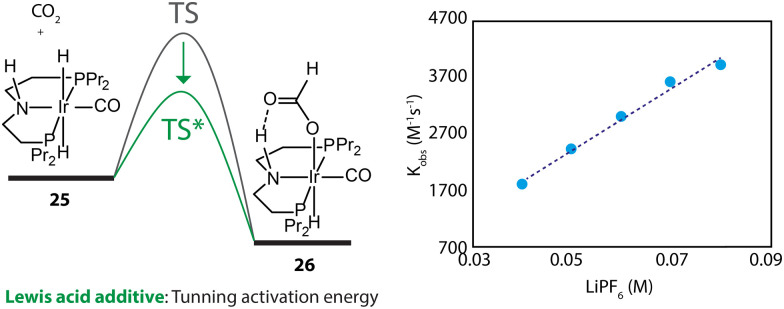
The dependency of the energy barrier for the hydride transfer process on Lewis acid concentration. The rate constants of the transformation 25–26 at different concentrations of LiPF_6_ in THF. Adapted with permission from ref. 111. © Licensed under a CC BY-NC 4.0 license.

Furthermore, the promotional effect of additives, that do not cause chemical changes, may stem from their perturbation to the reaction environment. For example, Lewis acids are widely used to promote the performance of CO_2_ hydrogenations.^110^ In the kinetics and mechanistic studies by Hazari and coworkers, the rate constant of the rate-determining step of an Ir-PNP-catalyzed system, hydride transfer from 25 to CO_2_, was found to be linearly dependent on the concentration of LiPF_6_ (Fig. 12).^111^ The Lewis acid in the catalysis medium lowered the activation energy of the hydride transfer and accelerated the overall reaction rate.

The change in reaction compositions can alter the reaction environment and redefine the kinetic and thermodynamic parameters of catalysis. Furthermore, the degrees of these intrinsic perturbations seem to be dependent on the concentration of the interferer component. This crucial feature readily distinguishes environmental effects from molecular interactions, which would show saturation at some asymptotic value.

As shown above, the effects of product formation on catalysis can only be extracted from reaction kinetics. Coupling this with the *in situ* tracking of the catalyst state with spectroscopy can further help determine which catalytic process is primarily perturbed, leading to the rational tuning of the system.

## 4. Catalyst deactivation

Catalyst deactivation is the least ambiguous parameter one can consider. It universally leads to the loss of the active species and the decrease of the catalytic performance.^112^ Due to the low concentration of catalyst and the co-occurrence of multiple deactivation pathways, mechanistic studies of deactivation in catalysis are challenging. However, understanding this process is still crucial for enhancing the stability of catalysts.

Although stabilized by substrate, hydrogen gas and other donor species, a hydrogenation catalyst can still degrade under the reaction conditions. For example, Beller and co-workers monitored the reaction mixture of Fe-^iPr^MACHO-catalyzed methanol dehydrogenation by NMR and found that the catalyst slowly decomposed by ligand dissociation, similar to the degradation pathway described for the base activation process (Fig. 7A).^113^

**Fig. 13:**
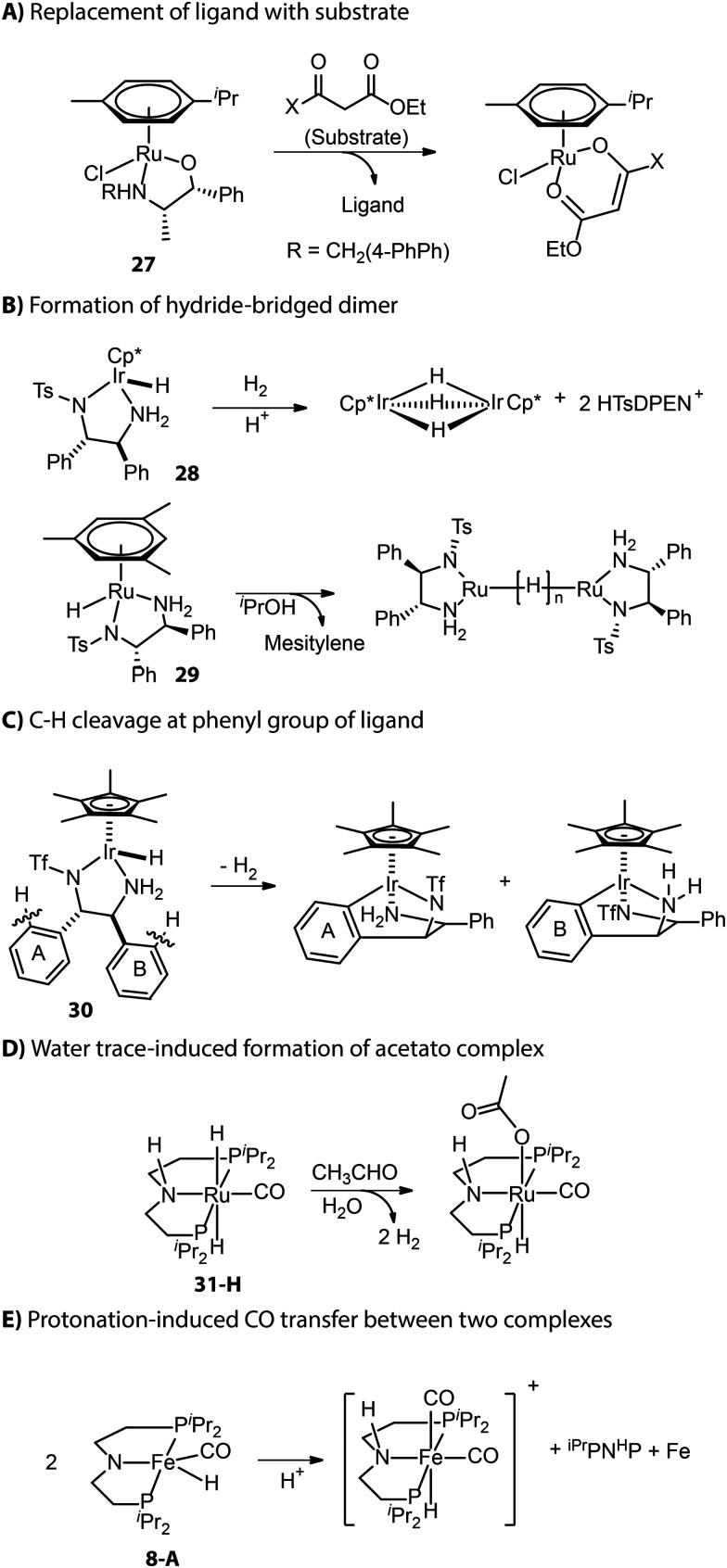
Deactivation pathways of hydrogenation catalysts.

Apart from those described in the catalyst activation process, new deactivation pathways occurring during catalysis have also been reported. As described by Carpentier and co-workers, the β-oxo ester substrate could act as a bidentate ligand and replace the ligand backbone of asymmetric transfer hydrogenation catalyst (β-amino alcohol)(arene)Ru (Fig. 13A).^114,115^ Inactive hydride-bridged metal dimers can form after the acid-induced or thermal dissociation of ligand from Ru or Ir center (Fig. 13B).^116,117^ The bifunctional Ir complex with the DPEN framework proved to degrade into iridacycles *via* C–H cleavage at the phenyl group of ligand backbone (Fig. 13C).^118^ The traces of water in the reaction medium could lead to the formation of inactive acetate Ru species from Ru-^iPr^MACHO in the dehydrogenative coupling catalysis (Fig. 13D).^119^ When amido Fe-^iPr^PNP was protonated by a bulky Brønsted acid, the resulting cationic complex could capture carbonyl from another complex and enter the inhibited state.^120^ Meanwhile, the other complex that provided the ancillary CO ligand would fully decompose to free ligand and iron nanoparticles (Fig. 13E).

**Fig. 14:**
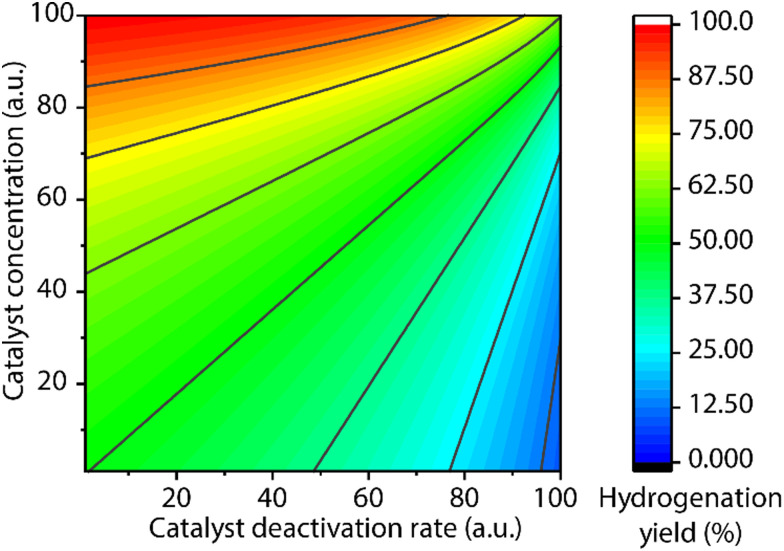
The competitive effects of initial catalyst concentration and catalyst deactivation rate on final reaction yield. The kinetic model of a catalytic ester hydrogenation reaction was used to predict the final conversions (the conversion at a very long reaction time) at variable initial catalyst concentration and catalyst deactivation rates (assumed as a first-order deactivation).

**Fig. 15:**
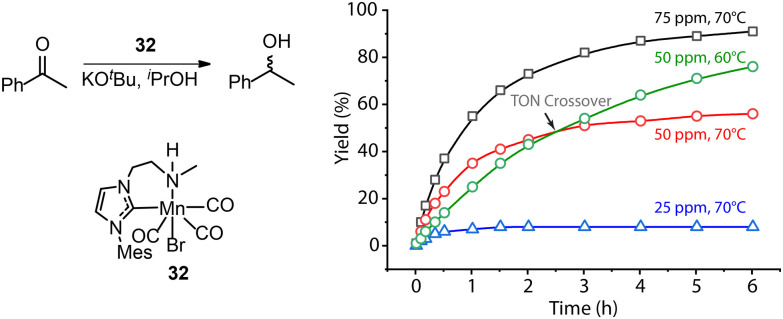
More noticeable catalyst deactivation at low catalyst loadings and high reaction temperatures. (Left) The transfer hydrogenation of acetophenons catalyzed by Mn-CN 32. (Right) The kinetic profile of the left catalysis at catalyst loadings of 25, 50 and 75 ppm and reaction temperatures of 60 and 70 °C. Adapted with permission from ref. 121. © Licensed under a CC BY-NC 4.0 license.

A common protocol to confirm the presence of catalyst deactivation is to check if a reaction gives non-quantitative conversion in prolonged reaction time, which is however not a robust test. Catalyst deactivation is not an instant process, and the remaining active catalyst species would promote the reaction before fully perishing. For some hydrogenation reactions, the increase of catalyst concentration can compensate the negative effect of the catalyst deactivation on the final catalytic results (Fig. 14). In general, full conversion in a catalytic reaction can be achieved as long as adequate amounts of the catalyst are added, which is not applicable when the deactivation rate of the catalyst is extremely high. For a good catalyst, its deactivation side-reactions feature a high energy barrier and progress at a relatively sluggish rate compared to catalytic turnover. Therefore, the deactivation can be easily overlooked and is significantly more noticeable at low catalyst loading. For instance, the transfer hydrogenation of acetophenone with 25 ppm Mn-CN catalyst 32 at 70 °C rapidly halted at around 1 h with 8% yield, while the catalysis with 50 and 75 ppm catalyst did not show signs of imminent termination (Fig. 15).^121^ The high energy barrier of the deactivation process can also render it extremely sensitive to temperature change. Namely, the rate of deactivation increases faster than that of catalysis when the temperature increases. In the same transfer hydrogenation system, the reaction at 70 °C was faster initially but was surpassed by the reaction performed at 60 °C with the same amount of 32 (50 ppm). This difference occurred due to the faster deactivation at 70 °C evident from the typical TON crossover point where a low-temperature reaction begins being more beneficial in terms of a number of turnovers compared to the high-temperature reaction.

Although catalyst deactivation is ultimately inevitable, it is up to researchers to pick the end to which the reaction comes – the catalytic performance can typically be improved by tuning the balance between deactivation and productive conversion. In the case of Ir catalyst 30, the removal of the phenyl group responsible for metallacycle formation (Fig. 13C) offered a new catalyst that achieved a 2-fold higher TON in the H_2_ evolution from formic acid for 125 min.^118^ For catalysts that suffer from reaction with water, *e.g.*31-H, the addition of molecular sieves or alkoxide base would allow for maintaining sustained catalytic performance.^119^

In addition, moving to pincer ligands from mono- or bidentate ones has been demonstrated as an effective strategy to counter catalyst deactivation. Indeed, for example, extending the bidentate ‘CN’ ligand of 32 with an additional phosphine donor led to a robust catalyst (Mn-CNP 1, Fig. 3A) that could tolerate high temperatures up to 120 °C.^55^ While the reduction rates with Mn-CNP were inferior to those with 32 at low temperatures, the improvement of thermal stability opened a broader temperature window for catalysis operation, among which enhanced catalytic performance was eventually achieved. This catalyst gave rise to an unprecedented turnover number (up to 200 000) in the hydrogenation of ketones at 120 °C.

## 5. Conclusion and outlook

We hopefully demonstrated that the catalyst performance is defined by a complex reaction network composed of multiple stages of catalyst operation. The rate and selectivity of pre-catalyst activation, the compositional effects imposed by the reaction environment and the catalyst deactivation dynamics all play a role in determining the apparent kinetics and the outcomes of catalytic hydrogenations. Studying these crucial parameters can provide direct instructions for improving catalytic systems. It can also help us comprehensively describe the complex reactivity networks so that the work put in catalyst optimization would progressively rely on more and more existing knowledge. Acknowledging the challenge of extracting such chemical information from the optimization studies with yield as the only descriptor of catalytic performance, we suggest that more data of kinetic and mechanistic nature need to be examined. While collecting massive kinetic datasets is burdensome and time-consuming on its own, a data-driven approach based on statistic modelling may offer the possibility of extracting kinetic information from high-throughput screening/optimization experiments.^122^

## Conflicts of interest

There are no conflicts to declare.

## Supplementary Material
